# Natural-Based Hydrogels for Tissue Engineering Applications

**DOI:** 10.3390/molecules25245858

**Published:** 2020-12-11

**Authors:** Manuel Gomez-Florit, Alberto Pardo, Rui M. A. Domingues, Ana L. Graça, Pedro S. Babo, Rui L. Reis, Manuela E. Gomes

**Affiliations:** 1 3B’s Research Group, I3Bs—Research Institute on Biomaterials, Biodegradables and Biomimetics, University of Minho, Headquarters of the European Institute of Excellence on Tissue Engineering and Regenerative Medicine, AvePark, 4805-017 Barco, Guimarães, Portugal; mgflorit@i3bs.uminho.pt (M.G.-F.); alberto.44444@gmail.com (A.P.); rui.domingues@i3bs.uminho.pt (R.M.A.D.); ana.graca@i3bs.uminho.pt (A.L.G.); pedro.babo@i3bs.uminho.pt (P.S.B.); rgreis@i3bs.uminho.pt (R.L.R.); 2 ICVS/3B’s—PT Government Associate Laboratory, 4710-057 Braga, Guimarães, Portugal

**Keywords:** biomimetic, extracellular matrix, proteins, glycosaminoglycans, decellularized tissue, DNA, blood derivatives, supramolecular crosslinking, nanoparticles, anisotropy

## Abstract

In the field of tissue engineering and regenerative medicine, hydrogels are used as biomaterials to support cell attachment and promote tissue regeneration due to their unique biomimetic characteristics. The use of natural-origin materials significantly influenced the origin and progress of the field due to their ability to mimic the native tissues’ extracellular matrix and biocompatibility. However, the majority of these natural materials failed to provide satisfactory cues to guide cell differentiation toward the formation of new tissues. In addition, the integration of technological advances, such as 3D printing, microfluidics and nanotechnology, in tissue engineering has obsoleted the first generation of natural-origin hydrogels. During the last decade, a new generation of hydrogels has emerged to meet the specific tissue necessities, to be used with state-of-the-art techniques and to capitalize the intrinsic characteristics of natural-based materials. In this review, we briefly examine important hydrogel crosslinking mechanisms. Then, the latest developments in engineering natural-based hydrogels are investigated and major applications in the field of tissue engineering and regenerative medicine are highlighted. Finally, the current limitations, future challenges and opportunities in this field are discussed to encourage realistic developments for the clinical translation of tissue engineering strategies.

## 1. Natural Polymers in Tissue Engineering

Tissue engineering promises to develop functional biological substitutes that restore, maintain, or improve tissue function using the principles of biology and engineering [1]. The field relies extensively on the use of scaffolds, cells and stimuli to induce new tissue formation. The incorporation of nature-derived materials as matrices or scaffolds to support and promote cell growth and proliferation has had a significant impact in the field of tissue engineering and regenerative medicine (TERM) [2,3].

Natural materials can be used to conduct multiple structural and biological functions due to their outstanding range of macromolecular designs, which have originated from the evolution of living beings in different environments throughout millions of years [2,3]. Among the different natural materials explored so far, the structural molecules are the most interesting for TERM purposes, being categorized into three different groups: i) proteins and peptides—amino acid chains (e.g., collagen, elastin); ii) polysaccharides—sugar chains (e.g., chitin, cellulose, glycosaminoglycans); iii) nucleic acids—nucleotide chains (DNA, RNA). Considering their source, natural materials might be originated from plants, animals (xenogenic) or humans (allogenic and autologous). Natural polymers offer several advantages with respect to synthetic polymers: 1) they present higher biocompatibility; 2) they can contain cell-binding motifs, enabling cell adhesion, spreading, and bioactive cues, as well as are able to influence cell behavior; 3) they can show a fibrillar architecture, mimicking the native tissues’ extracellular matrix (ECM); 4) they can be recognized and metabolically processed by the body, thus, allowing their remodeling by cells and concomitant deposition of cell-secreted ECM.

Hydrogels are three-dimensional (3D) networks formed by molecular chains embedded in a water-rich environment [4]. Hydrogels can be formed using several crosslinking mechanisms, show tunable physicochemical properties and high biomimicry of the native tissues’ ECM. In addition, hydrogels can be further modified with chemically and biologically active recognition cues such as stimuli-responsive molecules and growth factors (GFs) that enhance their biofunctionality [4,5,6]. Thus, due to the environment that hydrogels can provide, cells can grow, differentiate, and produce tissue-like structures.

Over the past decade, natural materials have gained momentum, provided by the engineering of a new generation of hydrogels intended to meet the specific tissues necessities and their use with state-of-the-art techniques. In this review, we will first introduce the different natural polymer crosslinking mechanisms and then discuss relevant examples of hydrogels developed using these polymers, specially focusing, but not limited to, 3D bioprinting applications. Then, the use of blood derivates and decellularized tissues as a source of biomimetic and bioinstructive natural-based hydrogels will be discussed. In the last section, the engineering of advanced hydrogels through the exploitation of supramolecular chemistry and nanotechnology is surveyed.

## 2. Hydrogel Crosslinking Mechanisms

**Figure 1:**
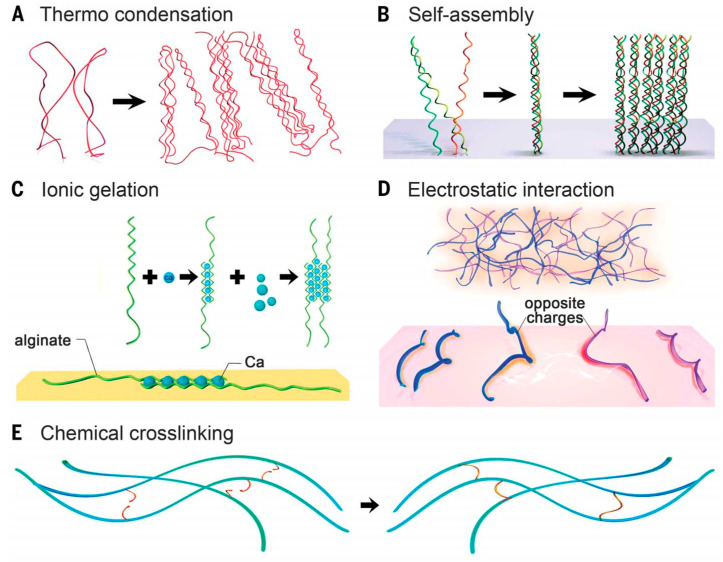
Crosslinking of hydrogels. (**A**) Thermally induced entanglement of polymer chains; (**B**) molecular self-assembly; (**C**) ionic gelation; (**D**) electrostatic interaction; (**E**) chemical crosslinking. Reproduced with permission from [7], copyright 2016 by American Association for the Advancement of Science.

Hydrogels are typically formed by crosslinking molecular chains dispersed in water-based solution through different mechanisms, including physical and chemical crosslinking (Figure 1) [7]. The physical gelation methods, including thermal and ionic gelation, self-assembly and electrostatic interactions, allows one to easily obtain hydrogels. However, the hydrogels produced using these methods have limited finetuning tolerance, as their characteristics are largely dependent on the intrinsic properties of the polymers. On the other hand, the chemical crosslinking methods allow a more controllable crosslinking but normally require the modification of the polymers, which can affect their biofunctionality.

### 2.1. Physical Crosslinking

*Thermo condensation*. It results from the physical entanglement of the polymer chains due to the temperature changes during the gelation process (Figure 1A). The increase or decrease in the temperature leads to a change in the polymer solubility that condensates, forming rigid polymer backbones. The gelation mechanism and transition temperatures are largely dependent on the polymer source and molecular weight [8,9]. A relevant example of this is gelatin. While gelatin from mammalian sources presents a higher gelling temperature (~25 °C), gelatin derived from cold-water fish has a lower gelling temperature (~10–12 °C) [10].

*Self-assembly*. It relies on the formation of weak noncovalent bonds between polymers (including van der Waals forces, hydrogen bonds, and hydrophobic interactions) (Figure 1B) [11]. Collagen hierarchical self-assembly is an example of this process that results from the regular composition of the amino acids proline and hydroxyproline in collagen molecules, which facilitate the formation of the collagen triple helix (also known as tropocollagen) [12].

*Ionic and electrostatic interactions.* These two physical crosslinking processes result from the opposite charges’ interaction (Figure 1C,D). Alginate is a well-known example of ionic crosslinking. Its gelation results from the chelation of divalent cations such as Ca^2+^ with its carboxylate moieties on the polymer backbone. The gelation by electrostatic interaction occurs between polymers that carry opposite net charges. Although many natural polymers possess a negative charge at pH ~ 7 because of carboxyl groups (such as hyaluronic acid and alginate), hydroxyl (e.g., gellam gum) or sulfate (e.g., chondroitin sulfate) groups, some are positively charged when amine groups dominate (such as alkali-treated gelatin or chitosan) [7].

### 2.2. Chemical Crosslinking

Hydrogels can also result from covalent bonding of polymers that possess chemically active motifs (Figure 1E). A myriad of strategies have been used such as carbodiimide chemistry between primary amines and carboxylic acids, aldehyde complementation, radical polymerization, high-energy irradiation, enzyme-enabled biochemistry, and click chemistry (e.g., thiol-vinyl sulfone and thiol-maleimide Michael addition reactions, azide-alkyne and azide-alkyne cycloaddition reactions), among others [13,14]. These methods promote a better hydrogel matrix stabilization when compared with physical mechanisms. Moreover, they allow a higher flexibility and spatiotemporal control of the hydrogel formation [7].

Due to its simplicity and reaction under mild conditions, enzymatic crosslinking has gained interest over the last years [15]. This strategy avoids some shortcomings associated with other crosslinking methods such as the potential cytotoxic effects induced by some chemical crosslinkers, their reaction conditions or by-products. Peroxidases, transglutaminases, and tyrosinases, among other enzymes, have recently been used to produce hydrogels for TERM applications. In particular, the reaction catalyzed by transglutaminases could be applied to any protein-based system since it catalyzes an acyl transfer reaction that yields cross-linked ε-(γ-glutamyl)-lysine isopeptide bonds between the abundant amino acids glutamic acid and lysine [16]. Additionally, hydrogels resulting from this crosslinking strategy are biocompatible and nonimmunogenic [16].

The use of electron irradiation to produce hydrogels has recently gained attention since it may provide high efficiency, high precision and fast crosslinking while not inducing cytotoxicity. Since this technique allows bulk as well as localized hydrogel crosslinking by using a highly focused electron beam, it introduces the possibility of patterning, actuation and sterilization, which may find many applications in TERM [17]. For example, collagen and gelatin hydrogels have been produced using this strategy, showing high cellular compatibility [17,18]. Nevertheless, this method requires very sophisticated equipment, not available in the vast majority of laboratories.

## 3. Hydrogels Inspired by the Extracellular Matrix

The ECM of native tissues provides physical support to preserve the structural integrity of tissues, serves as an adhesive substrate for the attachment and organization of cells, and as a reservoir for biochemical cues to support cell survival and differentiation [19]. Most ECMs are formed by a hydrogel-like porous network of fibrous proteins embedded in a soft matrix of glycosaminoglycans (GAGs) and proteoglycans. The ECM composition is tissue/organ-specific and correlates directly with tissue function. For example, while bone, as a load bearing tissue, is highly mineralized and stiff, tendons, responsible for transmitting tensile forces, are rich in bundles of elastic fibers. The tissue-specific ECM is deposited by the resident cell population in the tissue. Likewise, tissue-specific ECM features can induce the differentiation of progenitor/stem cells towards specific phenotypes [20]. Therefore, the use of ECM-derived natural polymers to produce hydrogels has a high potential to mimic the biophysical and biochemical features of the native tissues.

### 3.1. Proteins

Proteins derived from purified ECM or from recombinant sources, such as collagen, laminin, fibrin, fibronectin or elastin, have been used to develop biomimetic hydrogels and scaffolds for different TERM applications. Among them, collagen, accounting for nearly 90% of the dry weight of most tissues and organs (e.g., skin, bone, cartilage, tendon, and ligament), is the most studied protein of mammalian ECM for biomaterials preparation.

**Figure 2:**
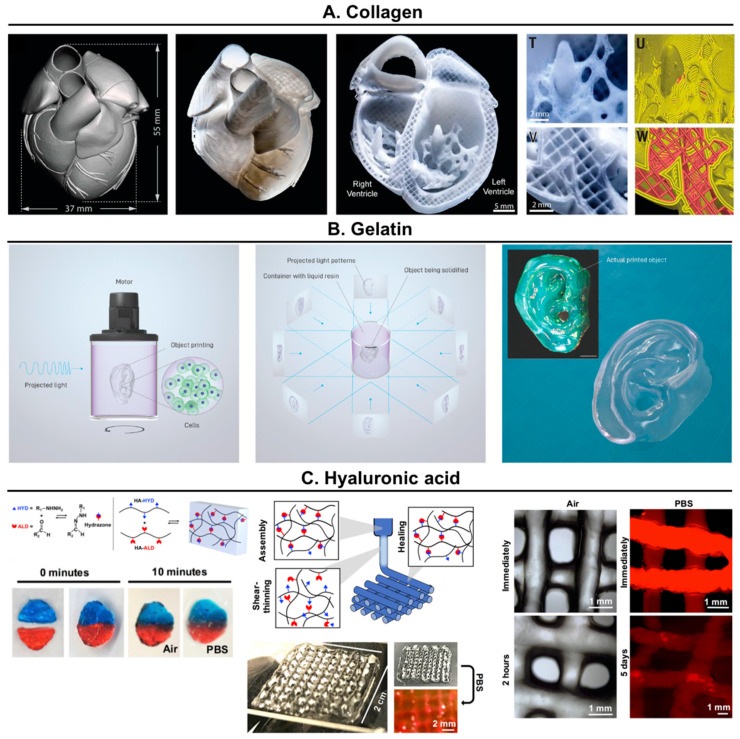
Examples of the use of natural proteins and polysaccharides for 3D bioprinting strategies. (**A**) Organ-scale 3D printing of collagen. Reproduced with permission from [21], copyright 2019, Springer Nature; (**B**) Volumetric bioprinting using gelatin with methacryloyl residues (GelMA). Reproduced with permission from [24] copyright 2019, The Authors; (**C**) Bioprinting of covalently crosslinked hyaluronic acid (HA) hydrogels. Reproduced with permission from [66], copyright 2018, Wiley Periodicals, Inc.

*Collagen.* It is a widely used ECM protein to produce hydrogels for biomedical applications due to its widespread availability in nature (bovine, porcine and marine), biocompatibility, biodegradability, and recognition by cells [12]. It can form gels by thermal condensation, although they show limited mechanical properties and stability, tending to degrade when cultured with cells. The mechanical properties of collagen hydrogels can be increased by chemical crosslinking (i.e., glutaraldehyde, formaldehyde, carbodiimide), by physical crosslinking (i.e., freeze-drying or heating dehydration), and by blending it with other polymers (e.g., alginate) [4,12]. In a very interesting work, Feinberg’s group relied on rapid pH changes to drive collagen self-assembly within a buffered support material (gelatin microparticles) [21]. This combination enabled the use of chemically unmodified collagen as a bioink to create bioprinted hearts that accurately reproduced patient-specific heart anatomical structures and showed synchronized contractions, directional action potential propagation, and wall thickening during peak systole (Figure 2A).

*Gelatin*. It is a partially hydrolyzed form of collagen, which can be easily processed and modified using different procedures and chemistries [22]. As explained, the thermal condensation of gelatin derived from many animal sources has been widely explored to form hydrogels. Regarding the chemical crosslinking of gelatin to produce materials, a good example could be the modification of gelatin with methacryloyl residues (GelMA), which has been commonly used to produce hydrogels with well-defined architectures using bioprinting, photomasking, micromolding, self-assembly, and microfluidic techniques. The obtained hydrogels have been evaluated as potential tools to regenerate different tissues (e.g., bone, cartilage, cardiac, vascular) as well as for drug and gene delivery [23]. Using volumetric bioprinting, an innovative bioprinting methodology based on visible light projection, GelMA, was used to bioprint clinically relevant sized, anatomically shaped, and cell-laden tissue-like structures with high viability (>85%), in short time frames (ranging from seconds to tens of seconds), compared with traditional 3D bioprinting technology (Figure 2B) [24]. Other strategies to generate injectable and covalently crosslinked gelatin hydrogels include, for instance the exploration of the enzymatic reactions catalyzed by transglutaminases, which produce stable hydrogels for various biomedical applications [16].

*Elastin.* It is a highly elastic ECM protein that allows tissues to repetitively and reversibly stretching or contracting. However, the use of elastin in the field of TERM is highly limited by its insoluble nature that makes its processing hurdling and incompatible with some techniques. Instead, tropoelastin, a soluble precursor of elastin, exhibits many physicochemical properties similar to those observed in elastin and is easier to manipulate [25,26]. Furthermore, tropoelastin has been shown to possess GF-like motogenic and mitogenic properties, promoting stem cell homing and proliferation [27]. Those features make tropoelastin a versatile molecule to engineer biomaterials with elastic properties. It has been explored as a bulk material to build highly elastic hydrogels [28,29], processed into films through casting [30], or electrospun fibrous scaffolds [31,32], which enhanced cell adhesion, proliferation and migration, and neo-vascularization. As well, it was used to produce surface coatings that promoted cell interaction with the interface and potentiated integration within the body [33,34]. More recently, the modification of tropoelastin with methacryloyl residues (MeTro) has resulted in biocompatible and highly elastic surgical sealant hydrogels that demonstrated complete sealing of severely leaking lung tissue in the absence of sutures or staples [35]. The blending of MeTro with other polymers has been explored to enhance their elasticity. For instance, it was blended with GelMA to obtain elastic bioinks that were used for 3D printing of vascularized cardiac constructs that showed endothelium barrier function and spontaneous beating of cardiac muscle cells [36].

Recombinant and synthetic elastin-like polypeptides (ELPs; repetition of valine-proline-glycine-valine-glycine (VPGVG) sequence), which mimic the extensibility and thermal properties of the naturally occurring molecule, have also been used to create a collection of biomaterials, including nanoparticles, electrospun microfibers and hydrogels crosslinked using different chemical strategies [37]. These ELPs might as well be modified to include “clickable” sequences (azides and alkynes) that react orthogonally to form irreversible covalent bonds, which result in hydrogels with amendable viscoelastic properties [38,39].

*Cell-binding protein domains*. Different polymers have been functionalized with cell-binding domains of ECM proteins to obtain bioactive hydrogels. The majority (89%) of the published studies from 1979 to 2018 utilized the arginine-glycine-asparagine (RGD) tripeptide, which is the minimal binding domain of various ECM proteins (e.g., fibronectin, vitronectin and laminin) to integrin receptors [40]. Beyond this, multiple cell-binding domains have been used for functionalizing polymers to enable cell attachment, migration and activation of specific pathways [40]. However, cell-binding domains show decreased binding affinity and specificity compared with the same domains presented within the native 3D protein structure [41,42]. Remarkably, RGD motif presentation in fibronectin sequences containing the major integrin-binding domain improved the binding affinity and modulated GFs signaling [43]. More recently, hyaluronic acid hydrogels modified with recombinant fibronectin fragments were designed to preferentially bind different integrins [41]. While hydrogels designed to bind α3/α5β1 integrin promoted the formation of a space-filling and mature vasculature, αvβ3 integrin-specific hydrogels or RGD-modified hydrogels promoted endothelial sprout clumping in vitro and leaky vessels in vivo. Hence, the presentation of cell-binding domains in their native 3D structural context might precisely control cell function and identify synergistic outcomes that enhance tissue regeneration.

*Peptides*. The use of peptides (short aminoacidic sequences) to create hydrogels provides several advantages such as biodegradability and biomimicry of specific structural and functional aspects of native ECM. These have been used to produce peptide-based hydrogels or conjugated or co-assembled with hydrogel-forming polymers. In particular, the supramolecular assembly capacity of peptide amphiphiles (PA), consisting of a charged hydrophilic head, a β-sheet forming domain and a hydrophobic alkyl tail (see a recent review by the Stupp’s group [44]), has been explored to create fibrillar structures with many applications in biomedicine [45,46,47,48]. PA can be as well co-assembled with hydrophobic polymer tails or polypeptides to construct self-assembled bioactive and biomimetic hydrogels [46,47]. Recently, a biodegradable self-healing polymer–peptide hydrogel made of a poly(γ-glutamic acid) network was physically crosslinked via conjugated β-sheet peptide sequences [49]. The mechanical properties of the hydrogels were tailored in the range of 10–200 kPa, which is in the region of many soft tissues, by altering the β-sheet peptide graft density and concentration. As well, hybrid PA-protein systems were used to produce complex hierarchical fibrillar membranes and 3D-printed constructs for TERM applications. First, hybrid PA-ELP served as building blocks to fabricate foldable bioactive membranes that guided the growth of endothelial and adipose-derived stem cells into tubular structures [50]. More recently, a range of ECM biomolecules (fibronectin, collagen, keratin, ELP, and hyaluronic acid) served as the protein component of the hybrid system, which were used as bioink (including adipose-derived stem cells) to bioprint complex hierarchical structures, showing that the combination of self-assembly with 3D bioprinting has a huge potential in the field [51].

### 3.2. Polysaccharides

GAGs are the polysaccharides most frequently found in the ECM of native tissues, typically conjugated with proteins forming the proteoglycans. These negatively charged linear polysaccharides play key structural and regulatory roles in the native ECMs and are involved in many cellular signaling processes with high impact over the growth and development of the different tissues. GAGs can be categorized in non-sulfated (hyaluronan) and sulfated (heparan sulfate, heparin, chondroitin sulfate, dermatan sulfate and keratan sulfate) that present very different characteristics in terms of function, structure and/or chemical composition. Considering the diverse cellular and biological functions of GAGs in native tissues, these have gained huge attention to develop biomaterials for the field of TERM [52,53].

*Chondroitin sulfate (CS).* It is a biodegradable sulfated GAG composed of repeating N-acetylgalactosamine and glucuronic acid units, possessing a high negative charge density. It can be found in the ECM of tissues such as bone and cartilage. The sulfation pattern and chain length of CS, mainly depend on the source, which makes CS very heterogeneous. The main CS sources for biomedical applications are cartilaginous tissues from bovine trachea and, in recent years, also from marine sources such as shark cartilage [54]. Due to its low molecular weight and anionic characteristics, CS has been leveraged for polyelectrolyte complexation with cationic polymers (mainly chitosan) to build, e.g., fibers, membranes and nanoparticles [55,56,57,58]. In order to form hydrogels, CS has been blended with other polymers and chemically modified using different strategies [52]. For example, Elisseeff and colleagues developed adhesive CS-based hydrogels by its chemical functionalization with both aldehyde and methacrylate groups, in order to form two functional arms: one arm to covalently bond to a biomaterial scaffold and the second arm bonding to the tissue surface [59]. These hydrogels (with and without cells) bonded to articular cartilage defects, which led to tissue repair in cartilage defects in vitro and in vivo. More recently, hydrogels composed of hyaluronic acid, gelatin and CS were synthetized via click chemistry (hyaluronic acid and CS were modified with 11-azido-3,6,9-trioxaundecan-1-amine while gelatin was modified with propiolic acid) [60]. These hydrogels supported the adhesion and proliferation of chondrocytes in vitro.

*Hyaluronic acid (HA).* It is the only non-sulfated GAG and consists of repeating disaccharide units of glucuronic acid and N-acetylglucosamine. It is the “backbone” of mammals’ ECM, having crucial roles in several cellular and tissue functions [61]. Although HA used for biotechnological and biomedical purposes is commonly isolated from animal source (mainly rooster combs), in recent years the industrial production of HA using microbial fermentation has become an excellent alternative [62]. Despite forming highly viscous solutions at low concentrations and being negatively charged, HA does not form hydrogels by any of the common physical gelation mechanisms (e.g., thermal, ionic). Nonetheless, it can be chemically modified in both its hydroxyl and carboxyl groups, which places it among the most used polymers to build hydrogels for biomedical applications [63]. Readers are addressed to excellent reviews from Burdick’s group to have a complete overview of HA-based biomaterials [63,64]. Among several strategies, the use of dynamic covalent bonds to form shear-thinning injectable hydrogels has gained attention in the field. They are reversible and in constant equilibrium between the bound and unbound state, while showing significantly higher strengths than physical bonds [65]. In a recent work, the use of hydrazone crosslinks between aldehyde-modified HA and hydrazide-modified HA resulted in shear-thinning and self-healing hydrogels that allowed the biofabrication of scaffolds using in 3D extrusion bioprinting (Figure 2C) [66]. In addition, using the same dynamic covalent crosslinking chemistry combined with thermoresponsive hydrazide-modified ELP and aldehyde-modified HA injectable hydrogels were also developed. These hydrogels allowed the encapsulated cells to maintain their ability to differentiate into multiple lineages after injection [67] and promoted an increase in the gene expression of cartilage markers and in GAG deposition while decreasing undesirable fibrocartilage phenotype [68].

### 3.3. Decellularized Tissues and Organs

The use of decellularization techniques to produce scaffolds was first published in 1948 [69]. However, their use is currently raising interest in the field of whole-organ engineering and in the development of in vitro models [20]. Generally, the organ decellularization protocol results in 3D scaffolds with intact native organ structure. The decellularized organ is subsequently recellularized with stem and/or progenitor cells, which are cultured in specific bioreactors to mimic the physiological conditions experienced by the specific organ [20]. Despite recent progress made in whole-organ engineering, the main challenges for clinical translation include the optimization of the decellularization and recellularization protocols, the formation of endothelial tissue, and the design of tissue-specific bioreactors.

On the other hand, a myriad of biomimetic hydrogels and scaffolds for diverse TERM applications have been developed from whole or purified ECM components (e.g., collagen, laminin, fibrin or fibronectin, as described in previous sections) [20]. ECM obtained from decellularized tissues (dECM) can be processed into biomimetic hydrogels through enzymatic solubilization followed by neutralization to physiological pH and temperature [70]. They can be obtained from any tissue, generating hydrogels with specific biochemical, architectural and viscoelastic characteristics depending on the tissue source and decellularization protocol [70]. These dECM hydrogels are biocompatible and have been used in preclinical applications, for example, as matrix for 3D cell culture, as media supplements, and as potential therapeutic agents for the treatment of various diseases [20].

**Figure 3:**
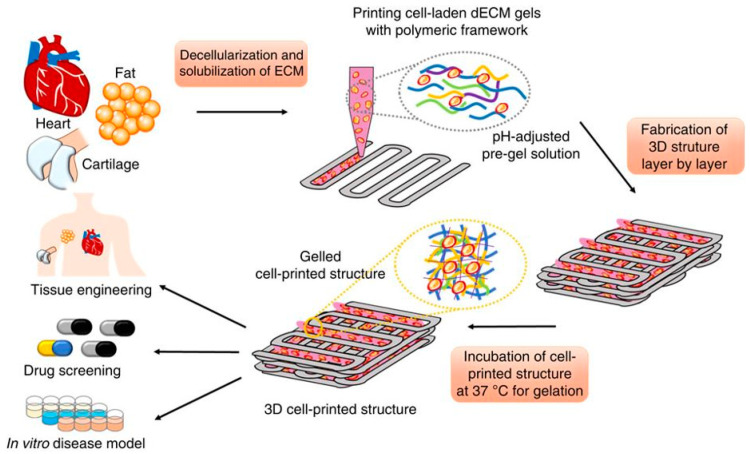
Representation of 3D printing using specific cell-laden dECM bioinks within a support framework. Reproduced with permission from [72], copyright 2014, Springer Nature.

Interestingly, dECM-based hydrogels have been suggested as promising materials to develop “game-changer” biomimetic bioinks for 3D bioprinting [71]. Multiple dECM bioinks have been produced from various organs and tissues such as heart, fat, liver, skin, cartilage, skeletal muscle, tendon and vascular tissue [71]. In a pioneering work, a bioink produced from heart dECM induced myoblasts to produce cardiac-specific proteins (actinin and fast myosin heavy chain-6), contrarily to a reference collagen-based bioink (Figure 3) [72,73]. Nevertheless, to allow typical layer-by-layer printing and obtain mechanically stable 3D constructs, these types of dECM bioinks have to be used at high concentrations, blended with other polymers or printed within supporting thermoplastics (e.g., PCL) frameworks. To avoid these limitations, dECM bioinks printing within microparticle support baths has been the state-of-the-art solution (readers are referred to a recent review about this methodology [74]). Using this methodology, pre-vascularized muscle constructs were fabricated through coaxial nozzle printing with muscle and vascular dECM bioinks [75]. The constructs successfully mimicked the hierarchical architecture of vascularized muscles and showed improved de novo muscle fiber formation, vascularization, and innervation, as well as 85% of functional recovery in a volumetric muscle loss injury rat model. Another elegant work inspired by the same methodology used omentum ECM to print thick and perfusable functional vascularized cardiac patches according to the patient’s anatomy and cellularized miniature human hearts [76]. Thus, dECM bioinks possess a huge potential to direct stem cell differentiation to create biomimetic and functional tissues, which would have applications in drug screening, disease modelling, and regenerative medicine.

Some of the advantages that dECM biomaterials exhibit over other hydrogels are the presence of organ/tissue specific biophysical- and biological- cues that potentiate the outstanding results on organ/tissue-like construct preparation ex vivo, as above presented, and their high clinical translation potential. Nonetheless, as dECM is produced either from human donations or animal explants, the immunological response, the high batch-to-batch variability and the limited availability of tissues need to be considered. In order to overcome these limitations, the use of cell cultures to produce dECM could offer a reliable alternative [20]. Using this approach, autologous cells could be cultured to obtain patient-specific dECMs that could avoid the immune response. Furthermore, these cell-culture derived dECM could be produced on a larger scale with higher reproducibility using cell expansion factories although it is still very far from clinical translation.

## 4. Nucleic Acid-Based Hydrogels

Deoxyribonucleic acid (DNA) is a very well-studied molecule composed by a double chain of four nucleotides paired with a specific complementarity, which plays a crucial role as genetic information storage in all live beings. Surprisingly, in recent years it has also become a key molecule in materials science. The remarkable capacity of sequences of nucleotides to pair with their complementary chain has been explored to prepare materials with programmable and multifunctional characteristics. Using single- or double-stranded DNA, hydrogels, aptamers and nanostructures have been recently developed for 3D cell culture, scaffolds for TERM and other biomedical applications [77,78,79,80]. DNA hydrogels can be produced by ligase crosslinking or by supramolecular self-assembly, enabling the gelling to occur under physiological conditions and, thus, the encapsulation of cells and biomolecules. These gels have shown excellent biocompatibility, biodegradation, molecular recognition properties, and nanometer-scale architecture [81]. In addition, given the excellent shear-thinning and self-healing properties of these hydrogels, they can be leveraged to develop injectable systems [79].

DNA secondary structures are very stable at physiological conditions although they are still reversible upon chemical, thermal, and mechanical stimuli. Thus, DNA hydrogels can respond to a variety of triggers, such as temperature, light, pH, enzymes or magnetic forces [78,80]. For example, a pH-sensitive DNA hydrogel able to undergo a conformational transition was able to influence the spatial distance between crosslinking points and subsequently the stiffness of the produced hydrogels. By increasing the pH from 5.0 to 8.0, the storage modulus of the hydrogels could be reversibly tuned from 1000 to 250 Pa [82].

**Figure 4:**
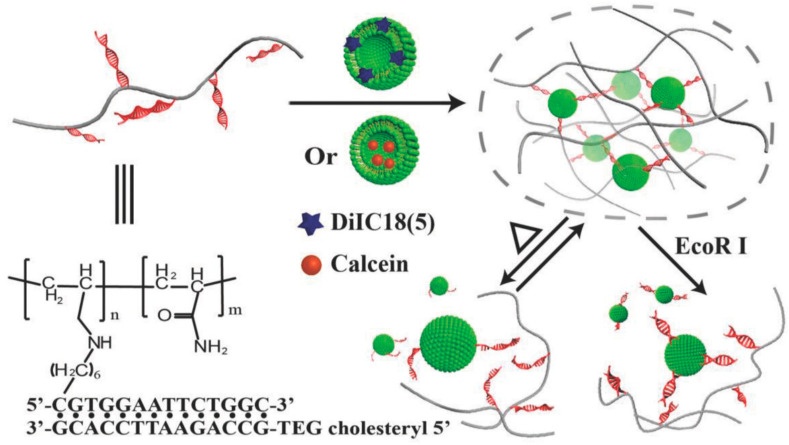
Schematic representation of the liposome–DNA hydrogel and its stimuli-responsive release behavior. Reproduced with permission from [85], copyright 2018, WILEY-VCH Verlag GmbH & Co.

Hybrid DNA hydrogels use synthetic or natural polymers as backbone to graft DNA sequences that are used as crosslinking points. Contrary to pure DNA hydrogels, hybrid hydrogels require little amounts of DNA, and self-assembled DNA acts as both the crosslinker and switchable element [78,79]. Li and colleagues grafted DNA to polypeptide backbones via azide−alkyne click chemistry, which allowed them to obtain a hybrid DNA-polypeptide hydrogel by hybridization with complementary linker DNAs [83]. These two-component hydrogels were loaded in a dual-nozzle printer, which enabled 3D bioprinting of constructs with high shape integrity and cell biocompatibility. Moreover, using the same hydrogels, the dynamic properties of DNA secondary structures were leveraged to obtain single component hydrogels with extraordinary self-healing properties [84]. In a different work, a stimuli-responsive liposome-DNA hydrogel was developed by the functionalization of polyacrylamide with cholesterol-modified DNA sequences (Figure 4) [85]. The cholesterol interacts with the lipid bilayers of the liposomes and crosslinks the polymer in a reversible manner (sol–gel–sol transitions) due to the thermosensitive nature of DNA motifs. Furthermore, the hydrogel can release the liposomes in an endonuclease-dependent manner.

DNA has also been used to develop aptamers or “nucleic acid antibodies”, short single-stranded oligonucleotides that have been widely evaluated as potential tools for disease diagnosis and therapy [80,86]. These molecules are designed to have high affinity degrees for specific target molecules using an in vitro selection and evolution process (also known as SELEX), making it theoretically possible to create aptamers that target any molecule of interest [87]. The generation of aptamers is much cheaper and easier than the generation of antibodies while minimizing ethical and immunogenic constrains, offering new perspectives in biomedicine. In an innovative work, DNA aptamers were designed to mimic the hepatocyte GF (HGF) receptor (also known as c-Met), which plays a key role in cancer metastasis [88]. It was able to inhibit HGF-induced c-Met activation, which suppressed cancer cell motility in vitro [88]. More recently, aptamers were engineered to bind platelet-derived GF (PDGF)-BB and vascular endothelial GF (VEGF) [89]. The two ends of the aptamer were linked to a functional group able to covalently link it to different materials (glass, polymers or collagen scaffolds) and to an RGD peptide, respectively. In this new strategy, exogenous triggers are not needed, being the specific GFs delivered as response to the cell traction forces on the ECM [89].

## 5. Blood Derivatives as a Source of Bioinstructive Hydrogels

Upon tissue injury, the provisional fibrillar matrix formed from blood components is necessary to achieve hemostasis, to enable cell infiltration, and to establish a spatial–temporal gradient of chemotactic proteins (e.g., GFs, cytokines, morphogens) that tightly regulates the complex wound healing process [90,91]. During normal wound repair, the blood clot is gradually degraded and substituted by mature ECM, following a series of sequential stages that culminate in the healing of the defect and reestablishment of tissue homeostasis. Blood derivatives, obtained from autologous or heterologous whole blood, have been extensively studied in TERM as a source of biochemical cues or structural polymers for the production of hydrogels to guide the wound healing process towards regenerative outcomes [91].

Blood derivatives obtained from citrated peripherical whole blood, namely, platelet-rich plasma (PRP) or platelet-poor plasma (PPP), are rich in fibrinogen and can be directly used as liquid-to-gel fibrin-based scaffolds through the activation of fibrinogenesis using collagen, calcium, or/and thrombin [91]. The properties of fibrin-based gels, such as fiber diameter, mechanical performance or bioactive molecules sequestration are strongly dependent on the dynamics of the polymerization processes. Therefore, they can be manipulated through variations in the concentrations of fibrinogen or the selected thrombogenic factor [92,93,94]. Fibrin-based hydrogels were early effectively translated to the clinics (e.g., fibrin glue during the 1980s). Throughout the last few decades, positive effects have been reported, for example, in the healing of periodontal and soft tissues [90,95,96,97]. However, the lack of standardized and well-characterized formulations, and conflicting therapeutic outcomes, have sustained the debate on their effective therapeutic potential [98,99].

Furthermore, the relevance of the fibrillar gels produced from blood-derived products, the richness in angiogenic GFs (e.g., VEGF, PDGF-BB, and transforming GF (TGF)-β1), particularly from the platelet-rich blood-derived products, has attracted significant interest to stimulate vascularization in tissue engineering strategies [100]. Nevertheless, besides the soft mechanical properties and high contractility upon cell encapsulation, the rapid turnover of the fibrin-based matrix of theses hydrogels limits the ability to protect GFs from fast clearance, which severely limits their use for TERM applications [101,102].

In order to overcome the aforementioned limitations, controlling the degradation of the hydrogel matrix, allowing the modulation of the spatiotemporal release of biomolecules delivery, and the reinforcement of the inherent mechanical and structural properties, different polymers from natural or synthetic origin and/or nanoparticles have been blended with blood derivatives [91]. Faramarzi et al. blended alginate with PRP (platelets suspended in PPP at 1×10^6^ platelet μL^−1^ concentration) to engineer a biofunctional bioink [103]. The developed PRP-based bioink exhibited a gradual release of bioactive proteins that enhanced mesenchymal stem cell (MSC) migration and human umbilical vein endothelial cell (HUVEC) tube formation capacity, constituting a promising biomaterial to induce a healing response in cardiovascular and musculoskeletal bioprinted tissue constructs.

Throughout the last few years, our research group has been developing different research lines based on the use of a particular type of blood derivative product, the platelet lysate (PL). It is obtained by the cryogenic disruption of the platelets contained in PRP, with a standard concentration of 1 × 10^6^ platelets/µL. The standardization of the protocol and blood derivative quality allows for higher consistency between batches [91,104]] and, therefore, in the properties of the derived biomaterials. We have shown that PL incorporation in HA hydrogels increased the stability of HA matrix and resilience to enzymatic digestion while allowing the sustained delivery of GFs over time [105]. Moreover, PL incorporation also enhanced the overall biological performance of the HA hydrogels, increasing the viability and osteogenic differentiation of the dental-origin MSC-like cells [106], and the survival of the human adipose-derived stem cells (hASCs) encapsulated into the HA/PL hydrogels during cryopreservation [107]. More recently, photocrosslinkable norbornene-modified HA (NorHA) was combined with human PL to obtain hydrogels that provided cell-anchorage sites and tailorable physicochemical properties, when compared to NorHA hydrogels [108]. Using microfluidic technology, PL-NorHA microgel spheres with a hierarchical fibrillar network were fabricated. These microgels could be jammed into granular hydrogels (read the following reviews for further information in granular hydrogels [109,110]) that exhibited shear-thinning and self-healing properties, enabling the fabrication of stable cell-laden 3D constructs with 3D bioprinting. More importantly, the incorporation of PL in the different systems improved HA-based hydrogels bioactivity.

Nanomaterials have also been explored to modulate the release of bioactive molecules from PL and to fine-tune PL-based hydrogels’ mechanical and biological properties [111]. This concept was explored to develop an injectable fibrillar nanocomposite hydrogel based on PL combined with cellulose nanocrystals (CNCs) grafted with aldehyde groups [112]. The induction of the coagulation cascade (using thrombin and calcium) together with the crosslinking between amine groups of PL proteins and aldehyde-CNC through reversible Schiff’s base bonds allowed us to obtain stable injectable fibrillar hydrogels. By increasing CNC content, it was possible to increase nanofibers and bulk hydrogels stiffness, modulate biomolecules sequestration, and impede the typical extensive hydrogel contraction upon cell encapsulation and culture. These characteristics enabled the modulation of encapsulated hASC growth, cytoskeleton organization and commitment towards different lineages. We further explored this hydrogel as a bioink for 3D printing applications [113]. Different complex structures were printed with high resolution, showing a fibrillar architecture from nano-to-macro scale. Additionally, this human-based nanocomposite bioink showed unprecedented levels of biofunctionality, allowing hASCs spreading and synthesis of their own ECM, compared with typical hydrogels used in the field (alginate and GelMA).

## 6. Engineering Advanced Hydrogels for Tissue Engineering Applications

The engineering of materials for tissue engineering applications is continuously assimilating novel methods and strategies from adjacent areas such as chemistry and nanotechnology. This has allowed the development of a wide range of hydrogels with more controlled physicochemical properties for the precise manipulation of cellular microenvironments. Recent developments have allowed the obtainment of hydrogels that show dynamic behavior, higher strength, controlled spatial architecture, and smart characteristics (e.g., materials that can respond to temperature, pH, magnetic, electric or any other external stimulus) [7,114,115].

### 6.1. Dynamic Hydrogels Based on Supramolecular Crosslinking

Usual hydrogels are typically formed using static crosslinking mechanisms that do not capture ECM highly dynamic character. In this sense, most static hydrogels are not able to dissipate stress, an important ability of native ECM originated by the reorganization of physical crosslinks or entanglements. Moreover, they also present important limitations in terms of the spreading, migration and proliferation of encapsulated cells [65]. Although hydrogel degradation may be mediated through hydrolytic or cellular enzymatic activity [116], the degradation of permanently crosslinked hydrogels presents several drawbacks, such as fast disappearance or deterioration and spatial heterogeneity of mechanical properties over time. These features are challenging to study and control how cells respond to local biophysical cues, which are increasingly recognized as key determinants of cell’s fate [117,118].

**Figure 5:**
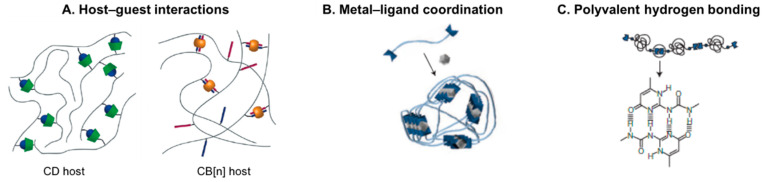
Supramolecular biomaterials formed by crosslinking of polymeric precursors through (**A**) host–guest complexation using macrocyclic hosts (**B**) coordination of metals with ligands (end-terminated or grafted on polymer chains), and (**C**) multiple hydrogen-bonding motifs. Reproduced with permissions from: (A) [11], copyright 2018, The Royal Society of Chemistry; (B) [135], copyright 2011, Nature Publishing Group; (C) [136], copyright 2005, Nature Publishing Group.

The use of hydrogels based on reversible (or adaptable) crosslinking mechanisms that can be broken and re-formed in a reversible manner (self-heal) under physiological conditions has attracted much attention in the design of biomaterials during recent years (Figure 5). Different comprehensive reviews widely covering this topic can be found in the literature [11,119,120]. The dynamic nature of the linkages used to obtain reversible hydrogel systems has noteworthy advantages over static crosslinking mechanisms to obtain hydrogels. This adaptability enables the spatiotemporally controlled addition and removal of biochemical signals and repeated variations in matrix mechanics. Furthermore, it also enables shear-thinning (viscous flow under shear stress) and self-healing (time dependent recovery upon relaxation) characteristics, which allow the encapsulation and delivery of cells using minimally invasive strategies [65,121]. This set of characteristics are also very interesting to develop bioinks for 3D bioprinting and to engineer complex tissue interfaces [119].

Among the possible methodologies to produce reversible hydrogels, supramolecular crosslinking of polymer chains based on host–guest inclusion complexes (Figure 5A), multiple hydrogen bonding or metal-ligand coordination (Figure 5B,C) are among the most attractive strategies [45,122].

*Host–guest interactions*. Polymer grafting with pendant host–guest moieties is one of the preferred strategies to obtain dynamic hydrogels. Among different systems, host macrocyclic molecules such as cyclodextrin (CD) and cucurbit[n]uril (CB[n], n = 5–8), which show high affinity for small guest hydrophobic molecules, are the most commonly used in biomedical applications [11,45,119]. Both bind guest molecules through hydrophobic and van der Waals interactions in the hydrophobic cavity that minimizes polar solvent interactions [11,123]. Natural (e.g., HA) and synthetic polymers (e.g., polyacrylamide or polyethylene glycol) have been grafted with either a host or its complementary guest molecule to form reversible supramolecular hydrogels when the pairs are mixed.

The host–guest pair, β-CD-adamantane, is another dynamic crosslinking able to develop injectable hydrogels that support cell encapsulation [119]. For instance, HA modified with these pendant motifs showed shear-thinning behavior that allowed injection and high site retention (>98%) [124]. In addition, the polymer network can be further stabilized through secondary Michael addition reaction (between HA macromers modified with thiol- and Michael-acceptor motifs). This injectable system showed positive results on the treatment of myocardial infarct using an in vivo mouse model. In a related approach, the low stability and mechanical properties of HA hydrogels crosslinked using the β-CD-adamantane pair were increased by first forming the host–guest complexes between monoacrylated β-CD host monomers and adamantane-functionalized HA guest polymers, to then fabricate the supramolecular hydrogels by UV-induced polymerization of preassembled host–guest complexes [125]. These hydrogels demonstrated self-healing behavior and showed promising results as injectable cell carriers, promoting chondrogenic differentiation of human MSCs and cartilage regeneration in a rat model. A representative example of the use of these materials as bioinks for 3D bioprinting has recently been developed by Burdick’s research group [126,127]. As the host–guest supramolecular bonds can be disrupted by the shear stress resulting from bioink extrusion during the printing process and rapidly reformed after deposition without any further trigger, this system allowed direct 3D printing at any position within supporting self-healing hydrogels made of similar materials. The printed structures can be additionally stabilized by secondary crosslinking by, e.g., methacrylation of HA and UV-induced photopolymerization post-printing. The versatility of this platform has demonstrated that dynamic hydrogels are potential biomaterials to be explored in the field of 3D bioprinting.

A different injectable and cytocompatible hydrogel has been formed based on HA modified with CB[6] mixed with the respective complementary guest, diaminohexane conjugated HA (DAH-HA), which was used to enhance chondrogenesis of human MSCs [128]. Interestingly, this host–guest pair was not only used to produce a cell carrier hydrogel, but also to functionalize HA backbone with a pro-chondrogenic drug conjugated to CB[6], showing the modular nature of these systems. The flexibility of this strategy was further explored to support the long-term survival and prolonged transgene expression of bioengineered MSCs, a potential 3D artificial ECM that could be applied for cell therapies and for the treatment of cancer and other diseases [129].

*Multiple hydrogen bonding*. Functionalization of polymers with hydrogen bonding moieties, such as the quadruple hydrogen bonding motif ureidopyrimidinone (UPy), is another widely reported strategy for the production of hydrogels showing viscoelastic, shear-thinning and self-healing behavior [45,120]. For example, dextran was functionalized with multiple pendant UPy units per chain [130]. The obtained supramolecular hydrogel was used as a cell carrier and drug delivery system (chondrocytes, bone marrow stem cells, and bone morphogenetic protein 2).

*Metal–ligand interactions*. This class of supramolecular crosslinking is based on the donation of a non-binding electron pair from two or more ligands to empty orbitals in a transition metal cation. It includes the mussel inspired Fe-catechol coordination complexes that show stability and strength values approaching covalent bonds [11,45]. Several hydrogels based on Fe-catechol crosslinking have been developed in the past few years targeting multiple biomedical needs [131]. For instance, HA modified with pendant catechol groups can be crosslinked with Fe^3+^ ions, resulting in reversible, self-healable and tissue-adhesive hydrogels [132,133,134]. These biocompatible injectable hydrogels enabled cell transplantation [133] and potentiated stem cell-mediated angiogenesis and osteogenesis in in vivo tissue defect models [134], making them promising materials for TERM applications.

### 6.2. Reinforced Nanocomposite Hydrogels

Conventional hydrogels typically possess a low to intermediate strength, which limits their application as load-bearing biomaterials. Alternatively, hydrogels reinforced with nanomaterials may possess substantially improved mechanical properties [115,137]. Nanomaterials (such as inorganic nanoparticles, carbon nanotubes, and cellulose or chitin nanocrystals) usually possess highly tunable surface properties and strong mechanics, which can contribute towards enhancing the chemical and mechanical performances of hydrogels.

The use of inorganic nanofillers has been widely explored for the mechanical reinforcement of hydrogels [138]. These include silicates (silica nanoparticles, bioglass nanoparticles and nanoclays) and calcium phosphates (hydroxyapatite, tricalcium phosphates), among others [138,139]. However, owing to the similarity of some of these particles to the mineralized native tissues (bone and interfaces), they have also been used to produce materials with superior biological performance [138,139]. For example, the incorporation of calcium phosphate nanoparticles, particularly hydroxyapatite (HyA), into hydrogel polymeric matrix has been explored for the production of osteogenic hydrogels for filling of small bone defects. HyA is the major component of bone mineral matrix and is osteoinductive and osteoconductive, being able to induce stem cell differentiation into osteoblast-like cells [140]. The incorporation in nano-HyA into gelatin cryogels increased the ultimate stress under compression from 98 to 524 kPa [141]. In addition, the nano-HyA particles enhanced the expression of osteogenic markers in MSCs in vitro [141,142]. Likewise, the incorporation of nano-HyA into chitosan hydrogels was shown to enhance the in vitro mineral matrix deposition and expression of alkaline phosphatase in MSCs, and promoted the healing of critical-sized defects in rat tibia [143].

The incorporation of different kinds of nanoparticles has also been evaluated as a potential strategy to develop bioinks that allow high printing resolutions and show high biocompatibility [144,145]. For instance, cationic silica nanoparticles were incorporated in alginate and gellam gum bioinks, observing a relevant improvement in the mechanical properties, printability, and printing fidelity due to electrostatic interactions between polymers and nanoparticles without affecting the viability of the encapsulated cells [146]. Addition of the nanoparticles (6 wt %) resulted in significantly increased shear thinning characteristics with much higher zero shear viscosity (2390 Pa s, 1062% increase) than the bioink without the particles (252 Pa s). In a different work, a synthetic silicate-based nanoclay with unique shear-thinning properties (Laponite) was incorporated in solutions of alginate and methylcellulose together with cells [147]. The designed bioink showed easy extrusion capability and allowed for the improvement of the shape fidelity of the printed constructs, also providing a tool for controlled release of GFs in comparison to the bioink without ceramic nanoparticles. Moreover, it improved cell adhesion, proliferation and human MSCs’ differentiation towards osteogenic lineages.

Cellulose nanocrystals (CNC), rod-shaped nanoparticles derived from cellulose (the nature’s “carbon nanotubes”), have shown great potential in TERM strategies [148]. Among different applications, CNC have been explored to crosslink and reinforce soft hydrogel networks. For example, hydrazone-crosslinked HA hydrogels were mechanically reinforced by aldehyde-CNC [149]. Hydrogels reinforced with different amounts of aldehyde-CNCs (0.125 and 0.25 wt %) exhibited storage modulus (E′) values between 107 and 152 kPa, which represented an increase by 65% and 135% compared with non-reinforced hydrogels. Overall, aldehyde-CNC incorporation in the HA hydrogel resulted in a more organized and compact network structure that led to stiffer hydrogels with lower swelling and higher resistance to degradation. More importantly, the cells encapsulated in HA-CNC hydrogels were able to spread and proliferate better than on HA hydrogels.

### 6.3. Smart Nanocomposite Hydrogels

Among multiple strategies to integrate smart functionalities in biomaterials, the inclusion of nanoparticles has become a popular option, not only as mere reinforcement nanofillers, but also as functional elements to achieve the needs of specific tissues [150]. These nanoparticles can be manipulated to alter different cell processes such as growth, differentiation, proliferation and alignment in a controlled manner [151]. In this section, we focus on recent developments in smart nanocomposite hydrogels based on natural materials.

The ability of magnetic nanoparticles (MNPs) to respond to alternating or non-alternating magnetic fields is highly appealing to produce responsive biomaterials [152]. In general, MNPs with superparamagnetic behavior are the most commonly used to develop hydrogels for TERM to preclude their aggregation as well as to avoid the generation of heat by magnetic hyperthermia (readers are addressed to an excellent review in the field [153]). However, there are many other parameters that are difficult to completely control when MNPs are incorporated into biomimetic materials, such as the particles’ colloidal stability in complex physiological environments, their precise localization/distribution and/or MNPs–biomolecules and MNPs–cells/tissues interactions [154]. Regarding the latter, the potential cytotoxicity of MNPs has been shown to be largely dependent on physicochemical properties, dosage and the way in which they interact with cells, that is, the mechanism and extent of cellular MNPs internalization [155,156]. The application of remote magnetic fields to hydrogels with magnetically responsive properties provides the liberty to control the 3D distribution and orientation of MNPs, which allows one not only to customize hydrogel’s internal architecture (reviewed below) but as well to remotely mechano-magnetically actuate the biomaterial. Actually, remote magnetic stimulation through magnetic responsive materials is emerging as one of the most interesting approaches to provide cells the adequate mechanical cues [157,158]. The effect of magneto-mechanical stimulation of neuron cells was analyzed using magnetic microparticles blended into high molecular weight hyaluronic acid hydrogels [159]. The resultant composites mimicked the mechanical properties of the spinal cord ECM, showing a storage modulus of 0.14 kPa. Moreover, the magnetic hydrogels facilitated the healthy growth of the encapsulated dorsal root ganglion neurons as well as the expression of excitatory and inhibitory ion channels. In this sense, the authors studied the effect of magneto-mechanical stimulation of the composites using a 2 T permanent magnet. While short-term “acute” stimulation enhanced the opening of endogenous mechano-sensitive ion channels allowing calcium influx, long-term “chronic” stimulation reduced the expression of these channels. Thus, these hydrogels are a versatile tool to study not only the effects of magneto-mechanical stimulation but, as well, a possible therapeutic strategy to reduce the expression of mechano-sensitive channels typically over-expressed in patients suffering from chronic pain. As well, magnetically responsive hydrogels have been used for minimally invasive TERM strategies. For instance, a hybrid hydrogel composed of type II collagen, HA, polyethylene glycol, and MNPs was guided to defect tissue sites using a remote magnetic field [160].

The incorporation of magnetic nanoparticles can be also explored to control the physicochemical properties of hydrogels overtime. In a recent work, we incorporated MNPs in a CS hydrogel loaded with PL and showed the ability to modulate hydrogel’s swelling, and degradation, while controlling the GF release profile [161].

**Figure 6:**
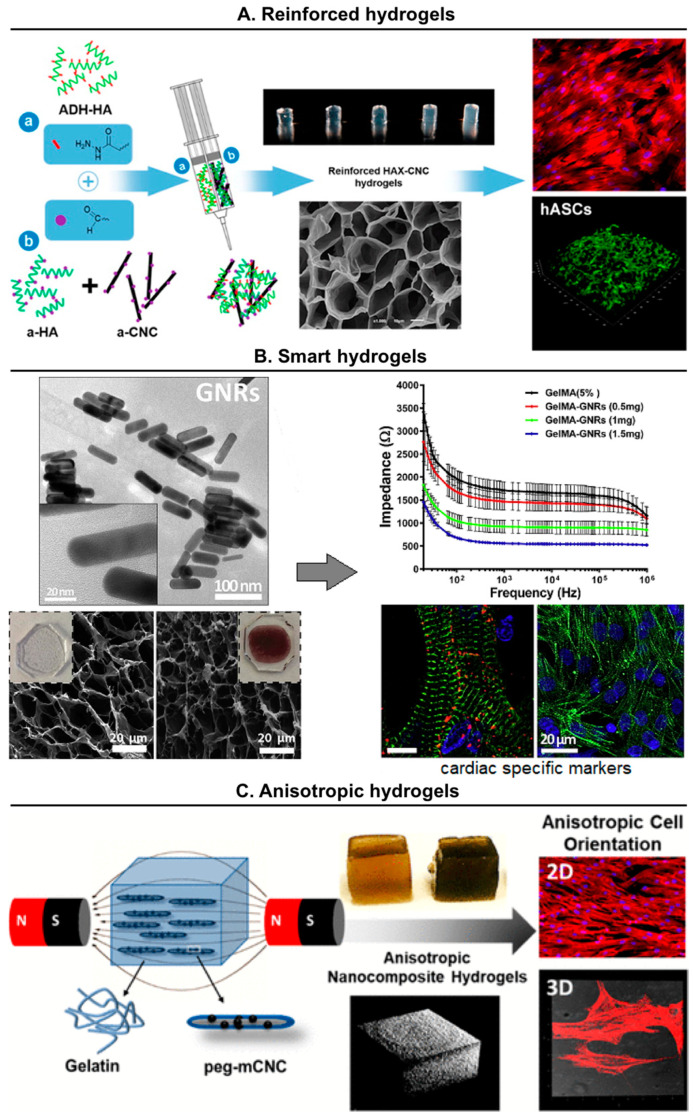
Examples of advanced nanocomposite hydrogels showing reinforced, smart or anisotropic characteristics. (**A**) Injectable hyaluronic acid (HA) hydrogels mechanically reinforced with cellulose nanocrystals (CNC). Reproduced with permission from [149], copyright 2015, American Chemical Society; (**B**) gelatin-based hydrogels with high electrical conductivity due to the incorporation of gold nanorods (GNRs). Adapted with permission from [166], copyright 2016, Elsevier; (**C**) anisotropic gelatin hydrogels produced by the magnetic alignment of magnetic-responsive particles (peg-mCNC). Reproduced with permission from [176], copyright 2019, American Chemical Society.

The incorporation of electroconductive nanoparticles can play an essential role in engineering strategies targeted to different organs/tissues with electro-responsive properties (heart, muscles and neural tissue) [162,163,164]. For example, nanocomposite hydrogels with electrical response were obtained through the inclusion of gold nanoparticles in chitosan matrices [165]. The presence of metallic nanoparticles increased significantly the electrical conductivity of the hydrogels. The MSCs seeded on these constructs showed high viability degrees and were able to migrate and proliferate, while showing evidence of accelerated cardiomyogenic differentiation in compassion with control hydrogels. In another work, rod-shaped gold nanoparticles were incorporated within gelatin-based hydrogels obtaining scaffolds with the suitable electrical conductivity and mechanical stiffness for cardiac tissue engineering [166]. The designed constructs improved the retention, viability and spreading of the seeded cardiomyocytes and promoted the homogeneous distribution of different cardiac specific markers (Figure 6B).

For a completely different purpose, in order to monitor cells within 3D bioprinted constructs, Trampe and coworkers developed an alginate bioink in combination with cytocompatible oxygen-sensitive nanoparticles. Using this system, the oxygen levels and the metabolic activity of different cell types were imaged online using optical sensors. This opens new opportunities towards the development of non-invasive techniques to map cells’ metabolism and to analyze their microenvironment in real-time after bioprinting [167].

### 6.4. Anisotropic Hydrogels

Hydrogels possess an inherent isotropic and disorganized internal structure, characterized by randomly oriented 3D networks, which limits their applicability in engineered oriented biological tissues [168]. This characteristic has promoted in recent years significant interest in the development of strategies allowing the modification of the hydrogel microstructure to provide them with the anisotropic organization required for the regeneration of anisotropic tissues (e.g., tendon, ligament, muscle, skin) [169,170,171]. Numerous fabrication strategies have been devised to create hydrogels with anisotropic architectures including the use of easily degradable components, gold nanoparticles or magnetic materials.

The preparation of hybrid hydrogels with easily degradable components to create anisotropic scaffolds has been extensively analyzed. Under this approach, it is possible to generate pores inside the hydrogels that allow the further growth and migration of cells. Nevertheless, a high control over the internal anisotropy degree is difficult to reach. Wang et al. [172], for instance, crosslinked GelMA in the presence of mesoporous silica nanoparticles, obtaining hydrogels with macroscopic pores up to 70 µm, but with a low control over their distribution inside the polymer matrices. In another approach, porous gradients were generated in a hydrogel composed of gelatin–hydroxyphenyl propionic acid (GTN–HPA) and carboxylmethyl cellulose–tyramine (CMC–TYR) conjugates [173]. The GTN–HPA component actuated as the hydrogel backbone, whilst CMC–TYR was used as biocompatible sacrificial polymer. The subsequent controlled diffusion of the cellulase enzyme selectively digested the CMC component, producing a porosity gradient on the hydrogel structure with a great control over the pore size, but not over their distribution inside the polymer matrices.

A more complex approach consists of the preparation of microgels and their further controlled assembly by different methods (bottom-up assembly). These procedures result in a better control over the hydrogels´ architecture than those provided by the use of porous/degradable components. In this way, Dinh et al. [174] added gold nanorods to GelMA microgels and used the intrinsic gold properties to guide the particles under near infrared (NIR) irradiation. By changing the size of the laser light spot, it was possible to control the assembly of the building blocks and the further crosslinking generated porous structures with the desired patterns for TERM applications. These low energy irradiations can be problematic in view of building human-sized tissues and organs, where they may not be energetic enough to ensure the desired penetration into the tissue engineered constructs.

A very recent and potential strategy to develop anisotropic hydrogels is the use of magnetically responsive elements in order to remotely control their spatial disposition through the application of external magnetic stimuli. For example, diamagnetic nanoparticles, such as CNC, and superparamagnetic particles, such as CNC decorated with MNPs, have been used to produce anisotropy within natural and synthetic hydrogels [175,176]. For example, we developed an enzymatically crosslinked gelatin-based hydrogel system, showing sections with distinct composition and architecture integrated in a single unit in order to recreate the interfaces of the musculoskeletal system, such as the tendon-to-bone transition (also known as enthesis) [177]. On one hand, CNC loading in gelatin and exposure to a uniform magnetic field (400 mT) before crosslinking resulted in anisotropic structures that allowed hASCs alignment and the deposition of ECM proteins related to tendon. On the other hand, the presence of hydroxyapatite microparticles generated stiffer hydrogels with higher ability to induce the differentiation of hASCs to osteogenic lineages. Moreover, when these CNCs were decorated with MNPs, the magnetic fields required to achieve the alignment of the nanoparticles were lower (106 mT) [176]. Moreover, the hydrogels showed 3D anisotropy, which induced the alignment of encapsulated and seeded hASCs, when compared with isotropic hydrogels (not exposed to magnetic fields).

## 7. Future Perspectives and Conclusions

Tissue engineering emerged with the promise of revolutionizing healthcare by providing artificially engineered functional tissue and organ substitutes. During the last few decades, extraordinary endeavors were initiated and significant scientific progress, ranging from cell biology up to advanced biomaterials synthesis and processing technologies, was achieved. However, the vast majority of strategies has been developed as “one-size-fits-all”. Biofabrication is an emerging and rapidly growing research field in which additive manufacturing has merged with tissue engineering to generate hierarchical tissue-like and personalized constructs. However, the development of bioinks that combine high resolution printability with cytocompatibility has been one of the major bottlenecks of the clinical translation of 3D bioprinting technology [5,145]. Thus, new polymeric biomaterials that can overcome these limitations are particularly needed, making reversible hydrogel systems appealing alternatives in this field. Moreover, another potential solution to address the lack of bioinks is the combination of a thermo-responsive gelatin network, which provides excellent extrusion and structural stability during 3D printing, and a photocrosslinkable network, which allows the printed structure to be stabilized by covalent crosslinking [178]. Furthermore, the dissociation of the thermo-reversible gelatin network does not affect the photocrosslinked network and supports 3D cell culture. Therefore, the use of polymer networks with complementary gelation mechanisms could be a broadly applicable methodology to expand the number of available bioinks allowing the biofabrication of constructs for TERM.

In addition, the extension of the concept of precision and personalized medicine treatments, in which healthcare is tailored on the basis of individual complexities [179], to TERM, will enable the engineering of biomaterials with precise and specific functions. In particular, the integration of precision medicine with the advanced biomaterials, technological advances such as microfabrication, 3D bioprinting and the new knowledge in stem cell biology, might allow for the selection of the most suitable approach to treat injuries or diseases in specific patients or groups of patients [180]. Furthermore, using the same principles, personalized tissue constructs possess a huge potential to become a new generation of disease models that substantially deviate from traditional platforms, namely, 2D cell monolayer cultures and animal disease models, changing the future of drug discovery pipelines [181].

The use of blood derivatives with biomaterials has appeared as a synergistic strategy to modulate the release of signaling biomolecules that orchestrate the swing between tissue repair, tissue regeneration and scarring [91]. For example, the use of biomaterials described in this review incorporating standardized blood-derived products combined with recent and future knowledge in the regenerative pathways, might enable one to modulate the wound healing microenvironment to promote tissue regeneration. In a clinical translation outlook, there is an increasing trend in the production and use of standardized clinical grade human PL as a xeno-free alternative to animal-derived serum in cell culture. This further strengthens the use of blood-product formulations, not only for research purposes, but also in terms of compliance with good manufacturing practices and clinical relevance, which is an advantage over other biomaterials such as dECM. Thus, this strategy holds huge potential to develop bioactive and biomimetic materials for different TERM applications.

Research in DNA-based materials is at the forefront of the biomaterials field due to its high versatility. In particular, the assessment of DNA origami structures for TERM applications is an under-researched area. These nanostructures could modulate cell behavior on 2D (surfaces) and 3D (hydrogels) environments. For example, the formation of DNA origami anisotropic patterns, as demonstrated in chemically modified graphene surfaces [182], could provide suitable biophysical cues to guide the adequate cells towards anisotropic tissues regeneration. Additionally, the capacity of DNA origami nanostructures to bind serum proteins, could be explored to selectively bind different GFs and to control its release to target tissues.

The majority of methods to achieve anisotropy within hydrogels depend on external magnetic or electrical stimuli, which is not practically scalable for large-scale production [183]. Hence, continuous developments in the area of soft nanocomposites are essential to produce scalable anisotropic structures showing excellent mechanical properties and to produce responsive materials to multiple stimuli for uses in innovative applications in the fields of TERM, biomedicine, actuators, and sensors. Moreover, injectable hydrogels with rapid self-healing and self-integrating properties may provide a novel, minimally invasive solution for the treatment and regeneration of tissues needing different spatiotemporal biochemical and biophysical cues but that seamlessly integrate at their interfaces (e.g., bone–cartilage tissue complex).

A new generation of living biomaterials containing genetically modified bacteria have been proposed as an out-of-the-box concept to dynamically modulate the biomaterials microenvironment. For this, bacteria are genetically modified to produce proteins of interest triggered by external stimuli such as molecules and drugs, in a dose-dependent manner [184,185]. Furthermore, the combination of technological advances such as biofabrication and this concept [186,187] might bring a new generation of living responsive biomaterials on demand. Nevertheless, the extended application of this concept would need to overcome serious bacterial safety concerns.

Despite the added functionalities that provide many new biomaterials, the clinical translation of TERM products has been significantly slower than would be expected. The complexity of some strategies represents the main hurdle for clinical translation, since economic and regulatory agencies favor simplicity [188,189]. Nonetheless, tissues and organs have hierarchical architectures, multiple cell types and ECM components, and complex vascular, neural, and lymphatic networks to support cell activity in a finely coordinated dynamic microenvironment. Balancing the need for simplicity with this natural complexity creates the necessity for the researchers of biomaterials to identify the strategies, within the large design space now available, with the minimum necessary complexity to recreate the native tissues.
